# Analysis of the Effects of Aggressive Environments Simulating Municipal Sewage on Recycled Concretes Based on Selected Ceramic Waste

**DOI:** 10.3390/ma11122565

**Published:** 2018-12-17

**Authors:** Bartosz Zegardło, Przemysław Brzyski, Katarzyna Rymuza, Antoni Bombik

**Affiliations:** 1 Department of Quantitative Methods and Spatial Management, Faculty of Natural Sciences, Siedlce University of Natural Sciences and Humanities, 14B Street Prusa, 08-110 Siedlce, Poland; bart.z@wp.pl (B.Z.); katarzyna.rymuza@uph.edu.pl (K.R.); antoni.bombik@uph.edu.pl (A.B.); 2 Department of Construction, Faculty of Civil Engineering and Architecture, Lublin University of Technology, 40 Nadbystrzycka Street, 20-618 Lublin, Poland

**Keywords:** recycling, ceramic waste, aggregate, concrete, aggressive environments

## Abstract

This paper presents the results of research aimed at finding the possible ways of disposing of ceramic waste material, focusing mainly on the possibility of using it as aggregates in concretes exposed to an aggressive chemical environment (municipal sewage). The research part presents the preparation method and investigation of waste ceramic aggregates (red, glazed and sanitary ceramic aggregates). A suitable ratio of coarse to fine aggregates was selected, and their density, absorptivity and crushing strength were examined. All examined aggregates were also subjected to SEM analysis. Red ceramic aggregate is characterized by a greater degree of crushing compared to glazed and sanitary ceramic aggregate, by 205.7% and 439.4%, respectively. Another part of the research was to compare the properties of concrete with traditional aggregate (gravel, basalt) and with ceramic waste aggregate. The tested parameters included consistency, apparent density, absorptivity, flexural and compressive strengths of concretes. The study proved that the absorptivity of recycled composites is higher than that of traditional composites by 20.8–24.7%. The concrete based on sanitary ceramic waste has the highest strength parameters. Its compressive strength is higher by 10.5% and flexural strength by 5.9% compared with the basalt aggregate concrete. The compressive strength of sanitary ceramics concrete is higher by 42% and by 59% compared with concrete based on glazed ceramic and red ceramic aggregate, respectively. The last part of the research was to examine the resistance of concrete to aggressive environment. The scope of the work included the preparation of the research environment in the form of solutions with an increased concentration of aggressive agents (hydronium, sulfate, magnesium, ammonium ions). Among the concretes with ceramic aggregate, the highest decrease in the compressive strength was demonstrated by the concrete based on red ceramics (128.2%), while the smallest was demonstrated by the concrete based on sanitary ceramics (aggregate from sanitary ceramics (15.4%). The mass loss at different time intervals and compressive strength loss of samples stored in solutions were tested. The smallest weight loss caused by aggressive environment attack was recorded in the concrete based on ceramic sanitary and glazed aggregate (20.2% and 34.5%, respectively, after 120 days of aggressive environment).

## 1. Introduction

The research on the reuse of waste substances carried out to date has brought many positive results, which have been successfully implemented for industrial activities. An example is fly ash being used as a pozzolanic material in concrete [1] or as a component of geopolymers [2]. Another industrial waste is sludge produced in sewage treatment plants. Suchorab et al. [3] used a sewage sludge to obtain lightweight aggregates for use in concrete. Also, agricultural waste is used for the production of building materials, for example, from industrial hemp cultivations. Brzyski et al. [4,5] used pieces of the wooden core of hemp stalks (hemp shives) together with lime binder to obtain a thermal insulating composite for use as wall material. The issue increasingly being considered in scientific communities is the rational management of the construction waste. This issue also concerns ceramic materials. The use of ceramic waste as a modifier and its addition to concrete dates back to ancient times. In ancient Rome, sometimes, crushed tiles and bricks in the form of powder were used as mortar components [6]. In the modern era, the aggregates from crushed building elements began to be re-used in concrete production after World War II. This was due to the large amount of debris remaining after war. The addition of ceramic bricks to concrete has even been regulated by the German standard DIN 4163: “Concrete with contrite bricks—specification of production and use” in 1951 [7]. The current research on the use of red ceramics in concrete is mainly focused on the ecological effect and also on the physical and mechanical properties of this ceramic waste concrete [6,8,9,10,11,12,13,14,15,16,17,18,19,20,21,22,23,24,25].

Gonzalez et al. [8] manufactured structural concrete using recycled brick aggregates and stated that it was possible to produce concrete characterized by medium strength using a high percentage of aggregates obtained from recycled brick. Gonzales et al. [9] investigated the effect of ceramic aggregates on the properties of high-performance concrete and showed that concrete produced with up to 30% fine red ceramic aggregates achieved similar or improved mechanical and durability properties to those of conventional concrete with natural aggregates. Utilization of waste ceramics as coarse aggregate in standard-strength structural concrete was also investigated by Anderson et al. [10]. Torkittikul et al. [11] used ceramic waste in the form of fine aggregate to produce Portland cement and fly ash concrete. He noticed that the compressive strength of the ceramic waste concrete increased with the ceramic waste content and was optimal at 50% for the control concrete. Improving the 28-day strength of concrete containing 75% of the ceramic waste aggregate as a substitute for natural aggregate was also noted by Awoyera et al. [12]. In turn, Cabrera-Covarrubias et al. [13] used fine ceramic aggregates as a partial replacement for natural sand in mortars and proved that the compressive and flexural strengths of the recycled mortars decrease proportionally to the amount of natural sand replacement used. The influence of replacing the sand with fine red ceramic aggregate was also studied by Silva et al. [14]. The results showed that the maximum proportion of ceramic aggregate that has a positive effect on mortar properties is 20%. A similar observation was described in [15], where replacing the traditional aggregate with ceramic aggregate (crushed roof tiles) in amounts above 30% resulted in a decrease in the compressive strength of the mortar. Vieria et al. [16] proved that as the content of fine red ceramic aggregate in the concrete increases, the concrete’s resistance to shrinkage decreases.

Other research results present the work carried out on the concretes made from precious white ceramic [25,26,27,28,29,30,31,32]. This type of material, obtained from the waste elements of sanitary or technical (e.g., electrical insulators) ceramic is important because of the beneficial effect on the concrete features [25,26,27,28,29,30,31,32]. The results show that the greater the addition of the ceramic aggregate, the higher the strength parameters of the concrete [29]. Interesting features of the concrete formed with the use of cullet sanitary aggregates, the composition of which is similar to the one of ceramic electrical insulators, are shown in paper [30]. To provide economic justification of the work, the authors look for special applications of this type of concrete. The results show that it can be successfully used under the conditions in which the concrete is exposed to high temperatures or in the places where a high abrasion resistance is required. Despite the fact that there has been no comprehensive experimental research conducted on elements made of concrete prepared with recycled precious ceramic aggregates, it is expected that this trend can be implemented in industry. Sanitary ceramic aggregates have also been used in the production of innovative mineral-asphalt mixtures [32].

Ceramic waste is also used in powder form. Kannan et al. [33] used it as a partial replacement of Portland cement, and stated that producing high-performance concrete could be an excellent source for recycling large quantities of ceramic waste powder. In turn, El-Dieb et al. [34] proved that the use of a 10% ceramic waste powder replacement (of Portland cement) level was adequate for strength improvement, and replacement levels between 10% and 20% could be used to improve the workability retention of concrete. Kulovana et al. [35] proved that the optimal amount of ceramic powder in the mix is 20% of the cement mass in the case of compressive strength, liquid water transport parameters and resistance to de-icing salts.

An important issue related to the durability of concretes is its resistance to aggressive chemical environments. Under the influence of external factors, concrete is often exposed to the destruction associated with the impact of harmful chemical compounds. Harmful environments may be related to the presence of gaseous pollutants (e.g., carbon dioxide, sulfur dioxide), mineral acids (e.g., sulfuric acid, hydrochloric acid) or organic acids (e.g., lactic or acetic acid) [36,37,38]. The chemical attack may vary depending on the environment to which the concrete is exposed. Aggressive environments could be, for example, acid rain, or pollution from chemical plants. The degree of building material deterioration is dependent on the acid concentration in the surrounding environment [39]. Municipal sewage also constitutes an aggressive environment. Among others, prefabricated concrete pipes are used for the discharge of sewage. Repairing corroded sewer pipes is a serious problem. The main source of deterioration is the effect of sulfides and hydrogen sulfide. The most dangerous compound in municipal sewage is H_2_S. After oxidation of this compound by aerobic bacteria, sulfuric acid is formed, which destroys concrete surfaces. [40]. There are certain techniques to improve the durability of concrete pipes, such as lowering the concentration of sulfide in wastewater by adding iron [41], making protective coatings on the concrete surface [42], or increasing the pH of the concrete surface by spraying with magnesium hydroxide [43]. Li et al. [44] proved that the resistance of concrete to the attack of sulfuric acid depends mainly on the amount of Ca(OH)_2_ produced during the hydration of the cement; therefore, it was proved [45] that the addition of pozzolanic components increases the resistance of the concrete to the sulfuric acid attack. Recycled aggregate is increasingly frequently used in concrete production (e.g., ceramic aggregates mentioned earlier). Therefore, there is a need to examine such concretes for chemical attack resistance, whether their presence will improve or degrade the durability of concretes. Higashiyama et al. [17] investigated the resistance of mortars with ceramic waste to chloride penetration and stated that the ceramic waste mortar significantly resists the chloride ingress in comparison with the river sand mortar. Gonzales et al. [9] proved that after 180 days of curing, the concretes produced with up to 50% content of coarse mixed aggregates (including waste from brick masonry and concrete) achieved low chloride corrosion risk. In turn, Medina [26] showed that chloride penetration was slightly deeper in recycled concretes based on sanitary ceramic aggregates in relation to concrete based on traditional aggregates. Vieira et al. [16] proved that in mixes with fine sanitary ceramic aggregates, chloride ion penetration is higher than in the case of concretes based on red ceramic aggregates, because of the more porous microstructure of sanitary ceramics. The resistance to an aggressive environment of the concrete with the addition of other aggregates from recycling was investigated by Thomas et al. [46]. These studies showed that the high-strength rubberized concrete is highly resistant to the aggressive sulfuric acid environments. Araghi et al. [47], on the other hand, tested the recycled concrete based on poly(ethylene terephthalate) (PET) particles and showed that its weight loss after immersion in 5% sulfuric acid solution decreases as the content of PET particles increases. Sofi [48] tested the durability of recycled concrete based on waste tyre rubber, and proved that the reduction in its weight and compressive strength after acid attack decreases as the content of waste tyre rubber increases. Yang et al. [49] proved that replacing the river sand with sea sand and fresh water with sea water results in the increase of the concrete’s resistance to sulfuric acid.

This work is a continuation of the previously conducted research, in which attention was focused on the waste ceramic matter obtained from sanitary ware products [26]. The article presents the waste ceramic products that have been proposed as a substrate for the industrial production of concrete for use in the production of sewer pipes for the discharge of municipal sewage. The suitable ratio of coarse to fine aggregates was selected, their density, absorptivity and crushing strength were examined. The aim of the research was to select specific types of ceramic construction waste (from three different kinds of ceramic products), determine their basic parameters, attempt to produce aggregate concrete, evaluate the basic parameters of the obtained concretes (consistency of fresh mix, apparent density, absorptivity, flexural and compressive strength) and evaluate the effect of modification of aggregate composition on the concretes resistance to aggressive environment.

The analysis of the results allowed the authors to identify the technical and environmental aspects of recycling ceramic waste material.

## 2. Materials and Methods

### 2.1. Aggregates

The selection of material for the study was made by segregating the waste deposited in illegal building material landfills. Among the ceramic waste, the largest percentage of waste included red ceramic waste in the form of crushed bricks, ceramic blocks and shards of roof tiles. Another chemical substance found among the other waste was ceramic floor and wall tiles—broken or in scraps. A less sizeable group consisted of sanitary ceramics fragments.

The aggregate used in the authors’ own research was collected from landfills and grouped according to the type and classification of ceramic waste into three groups, i.e., red ceramics, glazed ceramics and sanitary ceramics (Sed-bud, Siedlce, Poland). The same aggregates were also used in our own previous studies [50,51]. Sand, gravel and basalt (Sed-bud, Siedlce, Poland) were used as traditional aggregates.

Using an Energy Dispersive Spectrometer (EDS) detector Phenom G2 Pro (Phenom-World BV, Eindhoven, Netherlands), elemental analysis was performed. The chemical compositions of the aggregates used in the investigation are shown in Table 1.

As indicated in the experiment, the chemical compositions of the ceramic aggregates are very similar. In all ceramic aggregates, high amounts of SiO_2_ (over 54%), Al_2_O_3_ (over 24%), and K_2_O (over 3%) were found. High silica content also occurred in the basalt aggregate. Gravel contained mainly MgO (40.85%) and CaO (31.78%). The content of CaO was a feature of both traditional aggregates. There was no CaO in the recycled aggregates.

**Figure 1:**
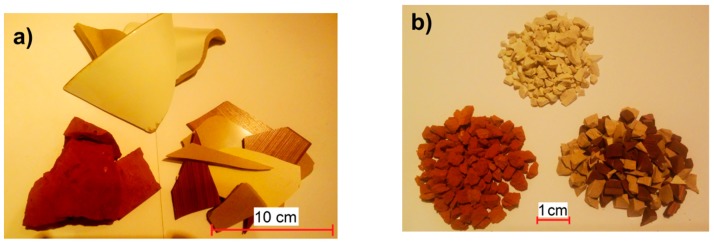
Ceramic waste materials. From the top: sanitary ceramic, red ceramic, and tiling ceramic, (**a**) in the form of waste, (**b**) in the form of coarse aggregate.

Thus, the obtained waste was subjected to crushing in MAKRUM 4015 jaw crushers (Makrum, Bydgoszcz, Poland). The use of the crushing machine enabled the segregation of aggregate grading into two types: fine grain size 0–4 mm and thick grain size 4–8 mm. Shards of ceramics with a diameter greater than 8 mm were placed in the crusher again. The ceramic waste generated from landfills and ceramic aggregate obtained by crushing are shown in Figure 1a,b.

A series of studies was carried out for the aggregate obtained this way, corresponding to the examination of natural aggregates that are commonly used in concrete [52,53]. Examination of particle size distribution, apparent and specific density and so-called crushing rate were carried out, as well.

#### 2.1.1. Particle Size Distribution

Obtaining the tightest plastic stack is beneficial to ensuring adequate load bearing. Therefore, the selection of grains in which the finer grains will tightly fill the spaces between the thicker grains is the goal. To determine the optimum ratio of coarse to fine grain size, a trial was carried out on the aggregates obtained from sanitary wares. Mixes of different proportions were poured into a dish with a volume of 1.69 dm^3^. By measuring the weight, bulk densities were calculated as well. 

The study on the grain composition of the aggregate mixture with this ratio was performed according to the PN-EN 933-1:2012 standard [54] after drying the aggregate. A mixture of aggregates of sanitary ceramic, glazed and red ceramics was subjected to trial tests. For each aggregate, a mixture of particle sizes in a ratio of 1:0.4 was prepared in an amount of 2000 g. After drying, the aggregate was subjected to sifting through a set of sieves. By weighing the portions of the aggregates which remained on the sieves, the percentage of content relative to the weight of the whole sample was evaluated.

The optimization test results are shown in Table 2.

**Figure 2:**
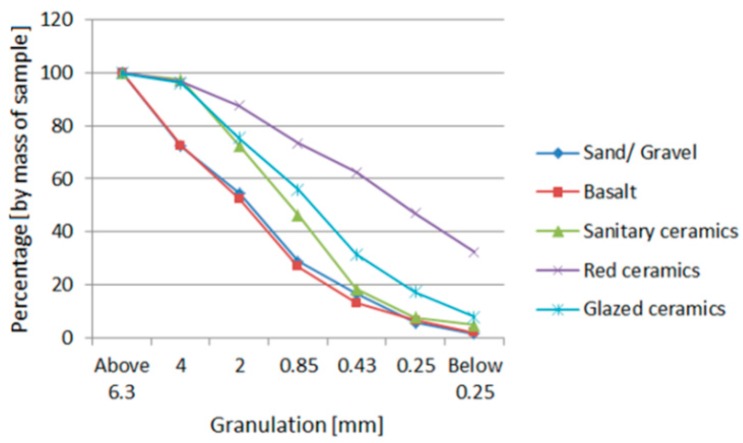
Grain size distribution curves for obtained aggregates.

On subsequent measurements, bulk density increased, and then it dropped. The point at which the bulk density was highest was considered to be the optimal ratio of fine aggregate to coarse, and this amounted to 1:0.4. The test results for the grain composition of aggregates mixture are presented in the diagram below (Figure 2).

The composition of grain size was not the same for all aggregates. Red ceramic aggregate contained many more fine grains than the other ceramic aggregates. The probable cause for this is that red ceramics have lower strength parameters. It was crushed by the jaw crusher to a greater extent than the other ceramic waste. The aforementioned supposition is confirmed by the degree of crushing test results. This parameter is much higher for red ceramics than for other aggregates. The test method for the rate of crushing and the process of crushing in crushers is similar. In this process, for aggregates of lower strength, during the crushing of large-sized material, it is possible to obtain a larger number of silt particles and particles of smaller diameters, but for the materials with higher strength, the particles will be greater in size. The different particle size distributions of the different types of aggregates can affect the concrete parameters. The effect of using aggregates with different particle size distributions in individual concretes will be a difference in density, which affects other properties, such as mechanical strength.

**Figure 3:**
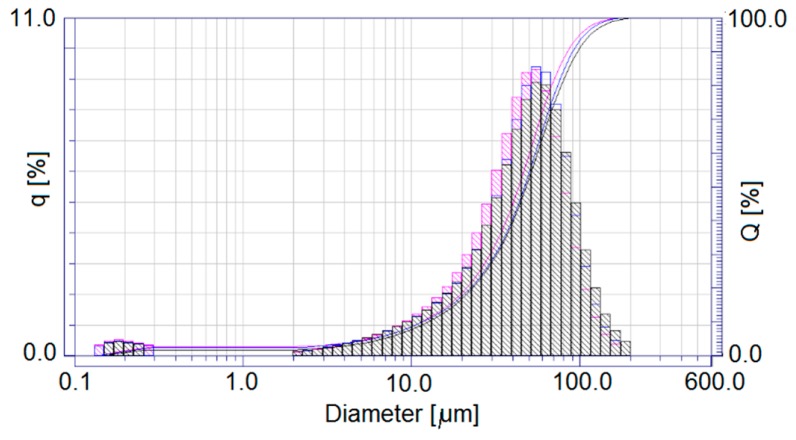
Particle size distribution curves for sanitary ceramic aggregates (*q* is the relative content [%]; *Q* is the cumulative content [%]).

The content of particles smaller than 0.063 mm was evaluated using Horiba LA-300 laser diffraction particle size analyzer (Horiba, Kioto, Japan) for a separated pile of grain size lesser than 0.25 mm. In the case of sanitary ceramics (Figure 3), the content of grains with a diameter lesser than 0.063 mm was 8.3%, which in relation to the total grain size of the aggregate was 0.372%. For other aggregates, the percentage of grains smaller than 0.063 mm in relation to the whole grain composition for glazed ceramics was 0.487%; for red ceramics, 5.336%; for basalt, 0.346%; and for gravel, 0.873%.

#### 2.1.2. Specific, Apparent Density and Absorptivity

The specific density was determined using the method provided in the PN-EN 1097-7 standard [55]. The test setup was equipped with a pycnometer with a volume of 50 ml (DanLab, Białystok, Poland), scales—RADWAG PSR2 (Radwag, Radom, Poland), laboratory dryer—SLW 15 (Pol-Eko, Wodzisław Śląski, Poland), vacuum pump—Basic 36 (Aga Labor, Warsaw, Poland) and a test sieve with a mesh of 0.125 mm (Multiserw-Morek, Brzeźnica, Poland). Nine measurements were made for each of the aggregates.

The apparent density and water absorption of the aggregates were determined according to the PN-EN 1097-6 standard [56]. From the aggregate samples weighing about 4000 g, grains with a size of 4 mm were separated, dried to a constant weight, and weighed. Afterwards, the grains were placed in water and left until complete saturation. The samples of aggregates in which water penetrated into the pores were removed from the dish and superficially dried so that their surface was wet, and water still remained in the pores. After making the appropriate measurements for weight, the absorption of the aggregates, expressed as a percentage of the mass of water which the aggregates were able to absorb to the dry weight of the aggregate, was calculated. On the same basis, the bulk density of the aggregates, as a ratio of the aggregate mass to its volume with the pores included in the aggregate, was calculated.

#### 2.1.3. Crushing Strength of the Aggregates

This test was performed in accordance with the PN-B-06714-40: 1978 standard [57] on the aggregates with a grain size of 4–8 mm. The test setup was equipped with a dish for measuring portions of the aggregate, laboratory scale and a sieve with a mesh of 1 mm. The strength test was performed on a Walter+Bai AG IF150 hydraulic press (Prüfmaschinen Testing Machines, Löhningen, Switzerland) using a special dish with a piston for crushing the aggregate.

The crushing indicator was established as a percentage (by mass) of grains that did not pass through a 1 mm sieve after crushing. Three tests for each type of aggregate were conducted.

### 2.2. Concrete

#### 2.2.1. Recipes

On the basis of the reference recipe for concrete C35/45, the main ingredient of which was basalt aggregate, a concrete was prepared in which the sand was replaced with crushed waste from ceramic of fine particle size, whereas ceramic aggregate of coarse grain size constituted the substitute for basalt grit. Aggregates were replaced by mass. In each formula, the weight ratios of the ingredients were the same.

Portland cement CEM I 42.5N/SR3/NA (Cemex Polska, Warsaw, Poland), was used as a cement in the concrete in accordance with PN-EN 197-1 standard [58]. It is characterized by stable physicochemical parameters, appropriate setting time, high early and final strength parameters, low alkali content and high resistance to aggressive chemical agents, which are commonly used in the production of commodity concrete mixes. The reason for using this cement also included its high resistance to aggressive environments declared in the technical sheet by the manufacturer. The ISOFLEX 7130 (Cemex Polska, Warsaw, Poland) agent was used as a plasticizing admixture. This admixture is manufactured using the latest hybrid polymers technology. This technology, using knowledge about the synthesis of molecules, enables a strong reduction of the amount of mixing water, long-term maintenance of the consistency of the concrete mix, as well as the homogeneity and cohesion of the concrete mix. Micro-silica (Ceta, Stalowa Wola, Poland) was used as a concrete additive. It constitutes finely grained dust consisting mainly of spherical, glazed grains. The silica fume used for the designed composites was obtained in the process of the gas cleaning of furnaces in the production of silicon-containing alloys.

Three concrete formulas were designed in which the aggregate was exclusively ceramic aggregate (red, glazed and sanitary), as well as two recipes using only traditional aggregates: gravel, sand and basalt. Despite the different absorbability of the aggregates used, the same W/C ratio = 0.37 was used in all formulas. The purpose of this was to determine the effect of only one variable factor (type of aggregate) on the tested concrete properties. The composition of the starting basalt concrete is shown in Table 3.

For comparison of results, besides the samples of basalt aggregate, the samples with gravel aggregate were prepared as well. Due to the fact that the largest dimensions of the aggregates in the presented concrete composites were 8 mm, the samples for strength tests were prepared in the form of beams with dimensions of 40 × 40 × 160 mm; in an amount of 9 pieces per recipe, in accordance with the literature data [52]. After forming, the samples were cared for under humid conditions. The samples were matured in accordance with the PN-EN 13670-1 standard [59]. For the first 3 days, the samples were stored in molds in a climatic chamber with a relative humidity of 95% and a temperature of 20 °C. Then the samples were demolded and matured in a relative humidity of about 40% in a laboratory room at a temperature of 20 °C ± 2 °C. The symbols of the tested recipes of concrete are shown in Table 4.

#### 2.2.2. The Consistency of a Fresh Concrete mix

The consistency was tested by concrete slump test in accordance with PN-EN 12350-2 [60]. The measurement for each recipe was carried out six times.

#### 2.2.3. Apparent Density, Absorptivity

The apparent density was tested for the samples with the dimensions of 40 × 40 × 160 mm. The tests were carried out on 6 samples from each recipe. The study was conducted in accordance with the PN-EN 12390-7 standard [61]. The apparent density was calculated as the ratio of the dry mass of the test samples to their volume. The absorptivity tests were conducted according to the PN-EN 13755 standard [62] on six 40 × 40 × 160 mm specimens from each of the recipes. The samples were totally submerged in the water. The results were the ratio between the mass of the absorbed water and the mass of the dry sample.

#### 2.2.4. Flexural and Compressive Strength

The samples were subjected to a flexural strength test by three-point bending, and then half of the trabeculae were subjected to the compressive strength test. The study was conducted in a Controls Advantest 9 testing machine (Controls Group, Milan, Italy) in line with the PN-EN 12390-5 [63] and PN-EN 12390-3 [64] standards. 

### 2.3. Simulation of Aggressive Environments

#### 2.3.1. Deterioration Mechanisms

Deterioration mechanisms vary depending on the type of factors that cause them. The adverse effect of acidic destruction applies to all concrete components, including aggregates (especially carbonate) [65]. It is induced by strong mineral acids (e.g., H_2_SO_4_, HCl, HNO_3_), weak acids (e.g., H_2_S) and organic acids (e.g., acetic, lactic and humic acids). Degradation is the formation of readily soluble salts as a result of the reactions described in the literature [66].

Acidic destruction leads to an increased porosity of the concrete and lowering of its strength. The mechanism of sulfate deterioration is based on the reaction of sulfate ions with the components of hardened cement paste. The corrosion products are insoluble compounds that attach to water in the crystallization process, resulting in a significant increase in volume. Initially, calcium hydroxide reacts by turning into hydrated calcium sulfate, in accordance with the reactions described in the literature [67]. In the next phase, a monosulfate mineral in the form of tiles is formed. In this phase, tricalcium sulfate (ettringite, Candlota salt) may also form in the form of clusters of elongated needles [67]. In the initial phase, sulfate corrosion has a beneficial effect on the concrete structure, because the reaction products fill the pores and capillaries, sealing the material and, as a result, increasing the strength parameters [68]. Further crystal growth causes high internal stresses, leading to scratches and cracks, and ultimately to total destruction of the material.

During magnesium deterioration, as a result of the reaction with calcium hydroxide, the magnesium ions replace the calcium ions. The product of the reaction is a slightly soluble magnesium hydroxide, which does not have binding properties, resulting in a weakening of the concrete structure. As in the case of the magnesium corrosion, the calcium ions are exchanged for the ammonium cations. The product are volatile ammonia and easily soluble salts, and as a result, the porosity of the concrete increases [68].

#### 2.3.2. Test Method

To imitate the chemically aggressive exploitation environment of pipes intended for the construction of municipal sewage systems, the concrete samples were placed in aqueous solutions of aggressive substances. Four types of aggressive agents were adopted, hydronium ions *H*_3_*O*^+^ (pH), sulfate ions *SO*_4_^2−^, ammonium ions *NH*_4_^+^ and magnesium ions *Mg*^2+^.

The work assumes a procedure for testing resistance to aggressive environments in accordance with the abovementioned literature [65,66,67,68] that describes the aggressive environments inside sewage pipes. Under standard operating conditions of sewer pipes, concrete deterioration is a long-term process. To obtain measurable symptoms of degradation in a shorter time, it was decided to accelerate its course by applying increased concentrations of aggressive factors. Ammonium and magnesium ions were introduced into the solution in the form of ammonium sulfate and magnesium sulfate. The composition of the solution is presented in Table 5.

**Figure 4:**
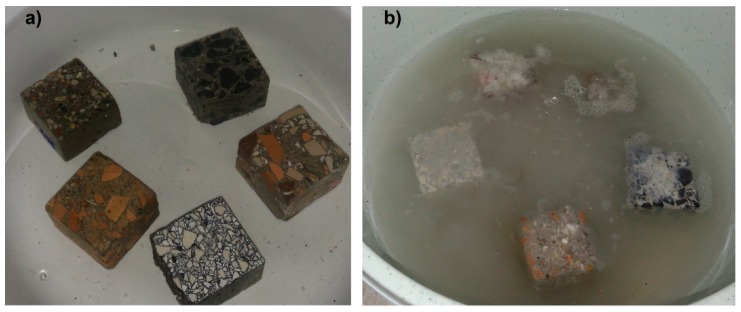
Samples of the tested composites on day 90 of the experiment (**a**) in water, and (**b**) in solution.

Test samples were placed in plastic containers containing the solution. For comparison purposes, some of the samples were placed in a container with tap water, containing no harmful agents. All samples of CC1, CC2, CC3, TC1, and TC2 composites for chemical resistance tests were cut out from the concrete samples with the dimensions of 150 × 150 × 150 mm. The samples were completely immersed in the test solutions, and their upper surface was about 20 mm below the table of the solutions to exclude the influence of hydrostatic pressure. The samples placed in research and comparison environments are shown in Figure 4.

The first criterion for assessing the resistance of composites to chemically aggressive environments was to test the compressive strength of samples after 40 days of immersing them in the solution. Another criterion for assessing chemical resistance was the loss of mass of samples caused by the solution. Before the start of the experiment, all samples were weighed in a dry state using a laboratory scale with an accuracy of 0.01 g. Subsequent measurements of mass change were carried out after 14, 30, 60, 90, 120 and 240 days. The research procedures (time intervals of measurements) were proposed by the authors. They resulted mainly from continuous observation of samples and visual assessment of samples during solution exchange.

## 3. Results and Discussion

### 3.1. Aggregates

#### 3.1.1. Specific, Apparent Density and Absorptivity

The test results were compared to the literature results [52] for the most common aggregates used in concrete plants and other recycling aggregates. The test results compared to the performance of the other aggregates are shown in Table 6. This table also presents other features of the aggregate with the values of parameters listed according to the test results available in the literature (ranges of compressive strength and modulus of elasticity) [52].

Table 6 shows the variety of a wide range of the tested parameters results. The reason for this is the quality of materials used for the production—i.e., clays—and the production processes. Due to the variety of applications, these features meet the technical requirements. However, these parameters will be very important in designing the process for concrete recycling. The aggregates with lower density and with a more open structure tend to absorb the slurry, which needs to be considered for each case in the design calculations. Recycled ceramic aggregates present relatively lower apparent density and higher absorptivity compared to traditional aggregates. Similar observations were presented in the literature [19]. Red ceramic aggregate is characterized by the highest absorptivity of 22%. A similar value, equal to 18.4%, showed the red ceramic aggregate with a fraction of 0–5 mm in the literature [69]. Sanitary ceramics is characterized by the highest quality in the case of all parameters. Similar values of density are presented in [29]. In other studies [31], sanitary ceramic aggregates were characterized by a lower absorptivity (0.2%), but with the bulk density of 2969 kg/m^3^. Ceramic sanitary wares with density of 2390 kg/m^3^, in turn, showed an absorptivity equal to 0.55% [26,27]

**Figure 5:**
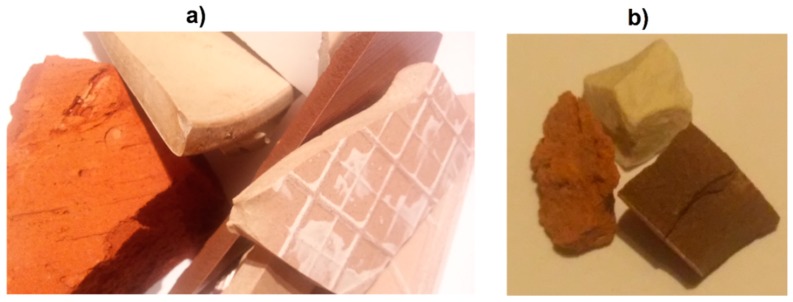
Close-up of the surface of the ceramic: (**a**) in the form of waste, (**b**) a form representative of the shape of aggregate grains.

The effect of high water absorption of the material and exposing it to the subsequent of freezing and thawing processed in the landfill were probably responsible for a significant weakening of the structure throughout the whole material. Due to the relatively low water absorption of other ceramic materials and the more coherent structure, the material was more unified, even after crushing. Figure 5a shows a close-up of the surface of waste ceramics, while Figure 5b shows a representative form of a particle shape of aggregates. The analysis of the surface of the grains formed after crushing proves that both glazed ceramics grains and sanitary ceramics grains were created as a result of brittle fracture. Their surface is smooth, and the edges are sharp and relatively aligned. The surface of red ceramic grain is characterized by numerous irregularities. The edges of the grains show numerous notches. These observations may confirm the thesis that the elements of red ceramics in jaw crushers are subject to crushing and not brittle cracking, as can be seen in the sieve analysis. 

**Figure 6:**
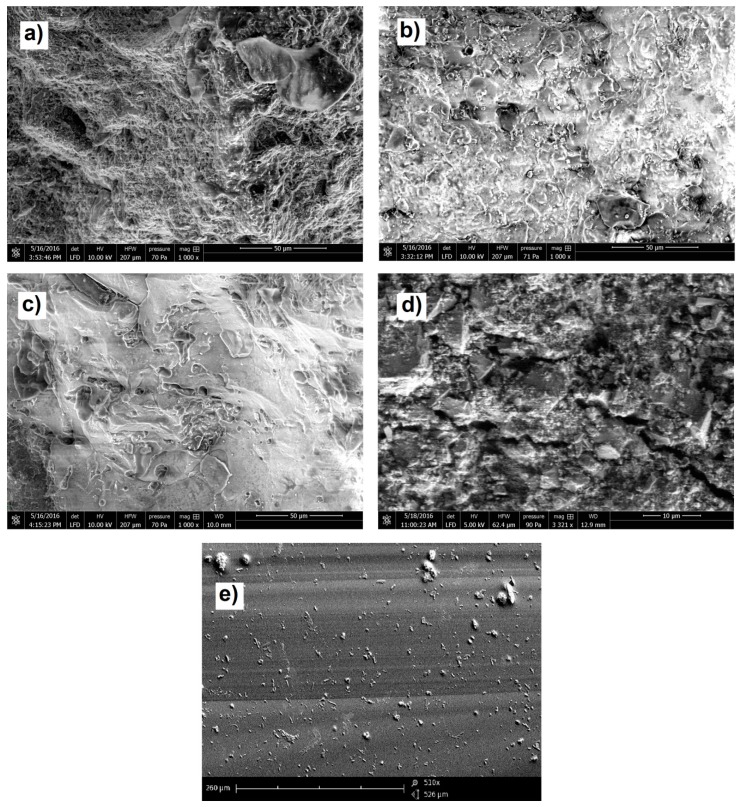
Images of aggregate grain structure: (**a**) obtained from red bricks (magnification 1000×), (**b**) glazed ceramics (magnification 1000×), (**c**) sanitary ceramics (magnification 1000×), (**d**) gravel (3321× magnification), (**e**) basalt (510× magnification).

Using the scanning microscope Phenom G2 Pro (Phenom-World BV, Eindhoven, The Netherlands), the structure of the surface of recycled aggregates and comparative aggregates was analyzed as well. The structure of grain surface is shown in Figure 6. 

On the basis of the presented scanning images, it can be observed that the structure of all the tested materials is very diverse. The image showing the surface of the red ceramics grain proves that this material has numerous pores, constituting the majority of its volume. This structure is the reason for the high absorbability of the red ceramics and their low mechanical strength.

Glazed ceramics also have numerous pores in their volume (visible on the surface); however, they are closer to the images obtained for sanitary ceramics. In both cases, the pores constitute a much smaller percentage of the material volume than in the red ceramics, while at the same magnification (1000×), a smaller number of them can be observed in sanitary ceramics. The “glassy” surface of both recycling materials is important. This effect is probably obtained by firing of clays at much higher temperatures, which causes melting of the material and its solidifying during cooling. This glazing may improve the technical parameters of aggregates, and may ultimately affect their increased resistance to chemical attack. Numerous pores in the space of both materials are the cause of their high absorbability.

The images of grain of comparative gravel aggregates indicate their irregular structure, the occurrence of numerous hollows, cracks and irregular pores. Such a structure causes their relatively high absorbability as well as low strength parameters. Basalt grains, on the other hand, show a smooth, homogeneous surface without pores, which explains their low absorbability and high strength parameters.

#### 3.1.2. Crushing Strength of Aggregate

**Figure 7:**
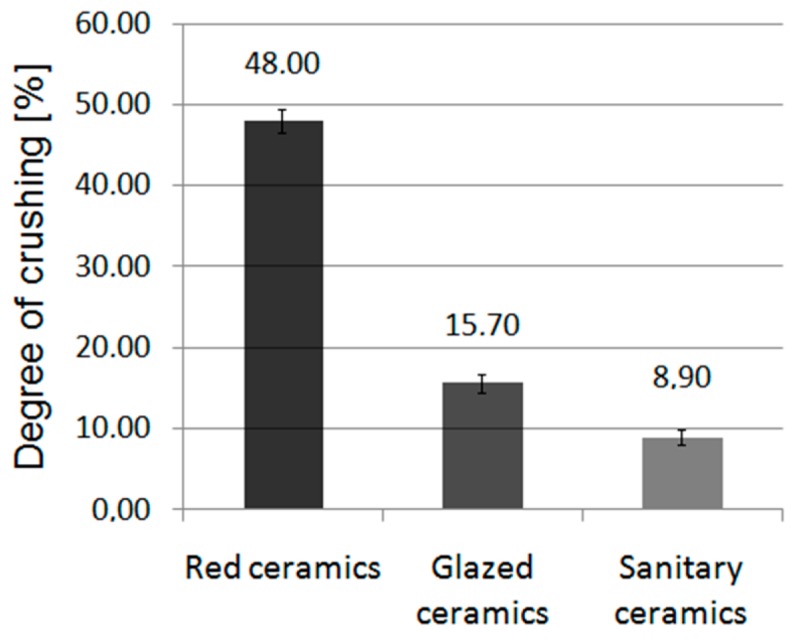
Degree of crushing of the tested ceramics (error bars mean standard deviations of degree of crushing).

The test results for crushing strength (average values) are presented in Figure 7.

Certain differences in the characteristics of aggregates can be analyzed during the crushing step. Sanitary and glazed ceramic materials behave similarly in the crusher; their fragmentation came in the form of brittle fracture, whereas the grains of obtained aggregate had a stocky form. Similar observations are described in [50]. Crushed red ceramic aggregate mainly shattered with crushing, and the aggregate grains had a flattened form. This result is due to the structure of materials. In the waste red ceramic products, a clear, layered structure of the ceramic material can be seen, where ceramic materials could be applied to each other in the process of preparing additional layers of material. Red ceramic aggregate is characterized by the highest crushing strength. In other studies [23], a crushing strength of 30.8% was obtained for red ceramic aggregates, with a density of 1805 kg/m^3^, and [8,9] 22.8% for masonry ceramics aggregates with an oven-dried particle density of 2000 kg/m^3^.

### 3.2. Concrete

#### 3.2.1. The Consistency of a Fresh Concrete Mix

The results of testing the consistency of concrete mixes are presented in Table 7.

The different consistency results with the same amount of water and plasticizer in all recipes proved that in terms of workability of the mixture, more favorable parameters were obtained using traditional aggregates. Ceramic aggregates are characterized by high open porosity and high absorptivity, which contributes to the absorption of certain amounts of cement paste. A smaller amount of cement paste surrounding the aggregate grain resulted in lowering both the fluidity of the mix and the consistency class. Similar observations are described in other studies [70]. Already after conducting this research, there were speculations that the contact zone of grains and cement matrix in the case of recyclable aggregates would be much more developed and coherent than in the case of traditional aggregates, which may in effect improve the strength parameters.

#### 3.2.2. Apparent Density, Absorptivity

The results of the apparent density and absorptivity of tested composites are presented in Table 8.

The results of the apparent density test showed that despite the same concrete components used (apart from aggregates), different values of apparent density of composites were noted. The differences within composites based on ceramic aggregate did not differ significantly between each other and this difference was about 2%. The apparent density of the composites containing traditional aggregates was higher, and for the composite with basalt aggregate, TC2 was about 14% higher than CC1. Cabrera-Covarrubias et al. [69] proved that the density decreases with the increase of the ceramic aggregate content relative to the traditional one. The differences were mainly due to the diversified aggregate density. However, there was no exact linear relationship between the density of the aggregate and the density of the composite. This was probably caused by the fact that the cement paste was absorbed by the aggregate. The pores filled with cement paste probably compensated for differences in the mass of the composite samples tested.

The absorptivity of the composites varied and, similarly to the density tests, the results were similar in the samples with ceramic aggregates. Vieira et al. [16] proved that the absorptivity increases with the increase of the ceramic aggregate content relative to the traditional one. The differences in the absorptivity of CC1 and CC3 composites were about 0.27%. The lowest absorptivity was recorded for the samples with basalt aggregate TC1 and it was 1.43% higher than the absorptivity of the composite based on red ceramics CC1. Considering the same quality of cement stone, in the case of absorptivity, the absorption of aggregates was the determining factor.

#### 3.2.3. Flexural and Compressive Strength

**Figure 8:**
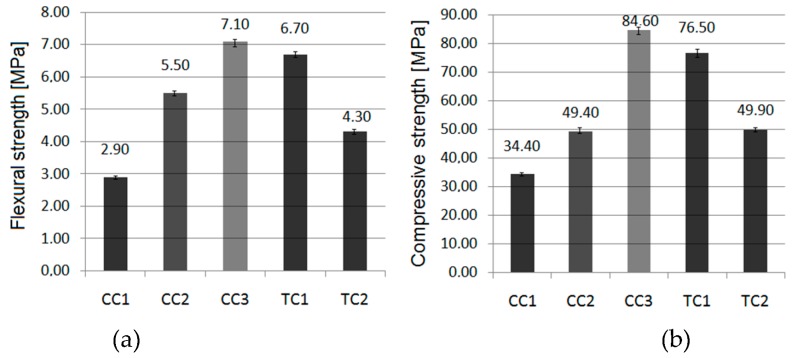
The strength test results: flexural strength (**a**), and compressive strength (**b**).

The average flexural and compressive strengths of the tested concrete samples are shown in the diagrams below (Figure 8). Error bars mean standard deviations of flexural and compressive strengths.

The flexural strength of ceramic concretes ranges from 2.90 to 7.10 MPa, and compressive strength ranges from 34.40 to 84.60 MPa. In addition to various aggregates, the particle size distribution of aggregates would be a source of differences in strength results. The results of concrete strength tests showed a clear improvement in the characteristics of the obtained concrete by replacing the original aggregate with the ceramic aggregate obtained from crushed ceramic sanitary waste. Similar observations are described in the literature [26,27,28,29,30]. Despite achieving a high performance for concrete containing crushed basalt (compressive strength 76.50 MPa, flexural strength 6.7 MPa) for recycled sanitary ceramic aggregate, the results were higher by 10.5% and by 5.9% for compressive and flexural strength, respectively (compressive strength 84.60 MPa, flexural strength 7.1 MPa). According to the literature [28], the presence of 9% sanitary ceramic aggregate increased the compressive strength of the gravel-based concrete by about 8%. Lopez et al. [29] proved that the use of white ceramic powder to substitute part of the sand gives an appreciable increase in compressive strength. Using white ceramic powder in a ratio of 1:1 with sand, the compressive strength increased by about 32%, compared to concrete based only on sand. The tested concrete containing entirely red ceramic aggregate had flexural and compressive characteristics that were relatively lower by 56 and 55% than concrete based on basalt aggregate. The decrease in the flexural strength (by 15–40%) and in the compressive strength (by 30–45%) after replacing gravel and sand with fine and coarse recycled red ceramic aggregate was also observed in the literature [19]. In other studies [22], together with increasing the proportion of recycled red ceramic aggregate in the composition of concrete (from 33 to 100%) the compressive strength decreased by 22–44%. Deterioration of the compressive strength of mortar based on crushed recycled brick aggregate in the amount of 10 and 20% was also observed [6]. On the other hand, Binici [24] proved that an improvement in strength occurs with the increase in red ceramics fine aggregate percentages (with bulk density of 1395 kg/m^3^). The concrete made with the participation of glaze aggregate had lower performance than a comparative concrete with basalt aggregate, by 17 and 35%, respectively. However, its parameters were almost identical to the parameters obtained for the concrete with gravel aggregate. The improvement in the strength of concrete resulting from the use of ceramic aggregate, compared to the usage of basalt aggregates, could be related to the quality of contact between the aggregates and the binder. The surfaces of basalt aggregates are smooth, and despite the fact that the aggregate is very robust during the strength tests, destruction occurs in places where the binder is connected to aggregate. Ceramic aggregates have the added advantage of an open microscopic structure [30]; there are numerous micro pores on the surface of the aggregate, which may be filled with binder. As a result, the contact surface is more developed, and in addition to the binder viscosity bonding to the aggregates, there can be mechanical bonds.

No relation was found between crushing strength of ceramic aggregates and the strength parameters of concrete, because red ceramic aggregates are characterized by the highest crushing strength, and concrete based on this aggregate has the lowest flexural and compressive strength among concretes based on ceramic aggregates.

Considering the results of the aggregate granulometry test and the content of grains smaller than 0.063 mm, a high content of dust in the aggregate may be the reason for low strength parameters of the composite with red ceramics. Covering the aggregate grains with dust hinders the access of cement paste to their surface. The result is that the grain-paste contact zone was weakened. The content of fine grains, with a diameter smaller than 0.063 mm, proved to be unfavorable from the point of view of concrete composite quality.

### 3.3. Resistance to Aggressive Environments

**Figure 9:**
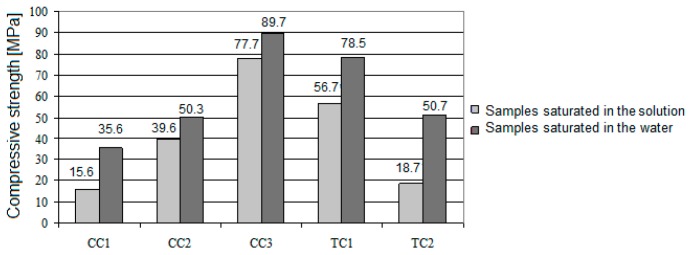
Averaged values of compressive strength of composite samples stored in water and aggressive environments.

The results of the compressive strength test of concrete samples exposed to a chemically aggressive environment are shown in the graph below (Figure 9). The values tested for the samples stored in the water were used as comparative values.

For all the tested samples immersed in an aggressive environment, lower strength values were observed than for the samples immersed in water. The form of destruction of the samples also showed the effect of aggressive environment. All tested samples were destroyed in the first phase by splitting off the surface layers directly affected by the solution. The lowest values were found for the samples CC1 (15.6 MPa) and TC2 (18.7 MPa). The highest strength was exhibited by the sample CC3 (77.7 MPa). Compared with the strength of a high-quality basalt aggregate composite (56.7 MPa), the strength of the composite made of sanitary ceramics was 27% higher. The decrease in compressive strength of basalt concrete and concrete based on sanitary ceramic aggregate was 27.8% and 13.4%, respectively. After 40 days of testing, the cement matrix weakened in the samples (aforementioned chipping of the surface layers during the compression test), which appeared to be particularly intensive in the case of the composites with non-porous aggregates (basalt). As a result of the weakening of the cement matrix, the adhesion force of the aggregate grains to the matrix weakened. In this case, the mechanical bonds between the cement matrix and the pores of the open construction aggregates proved to be more advantageous. The analysis of loose grains from the surface layers of basalt aggregate during destructive tests proved that the bond ceased to exist (the weakened paste was washed out under the stream of water). In the case of the ceramic aggregate grains, the cement paste remained in the pores of the aggregate. Weakening of the structure of composites based on red ceramics and gravel was caused by the fact that after 40 days of the experiment, the solution started to have a destructive effect on the aggregate grains. For comparison, in other studies [71], the reduction in compressive strength of recycled concrete (based on concrete waste aggregates) cubes after 30 days of immersion in 3% H_2_SO_4_ was about 43.86%. Gireesh [72], in turn, proved that loss in compressive strength of recycled concrete (based on aluminum dross aggregates) mixes due to acid attack (5% H_2_SO_4_) was in the range of 1.78–5.11%.

**Figure 10:**
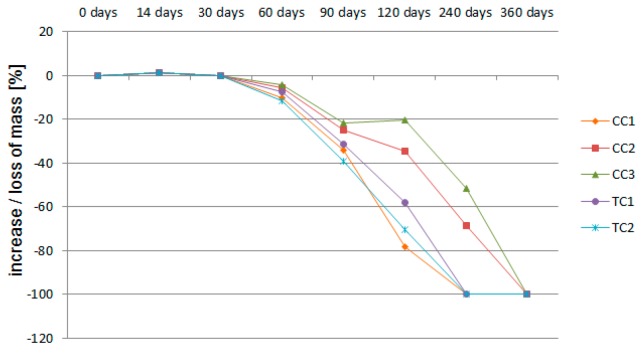
Changes in mass of samples.

The results of the measurements pertaining to the percentage change in mass of concrete samples are presented in Table 9 and in the graph (Figure 10).

In the initial period of the experiment, mass gain was observed in all of the tested composites, which resulted from the appearance of crystalline reaction products on the surface of the samples. Then, from the 14th day after the experiment started, decreases in the mass of the samples caused by aggressive environment attack were recorded. Already in this period, exposed aggregate was observed on the surfaces of all samples. During the analysis of the results, it was found that the fastest mass losses occurred in the samples with gravel aggregate and red ceramics. The weight drops that progressed the least were observed in the composites with sanitary and glazed ceramics. 

**Figure 11:**
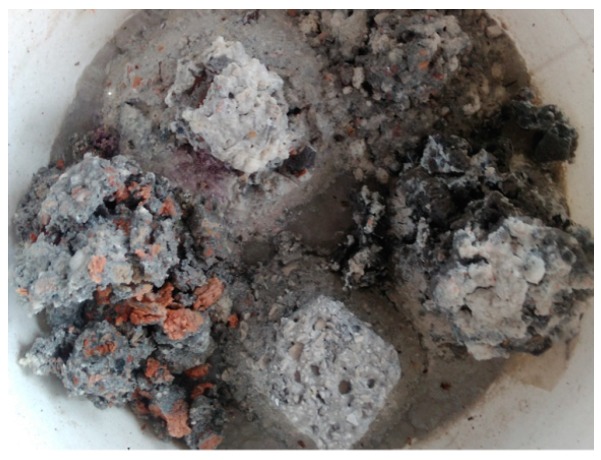
Samples of composites after 240 days of saturation in the solution.

The mass decreases of the samples recorded after 120 days of immersion in the solution were chosen as reference values for the comparison purposes. The weight loss of the CC1 composite was 78.4%. The CC1 sample was destroyed to such an extent that only its core remained. A similar observation was found for the TC2 composite sample. The weight loss value of this sample was 70.6%. The samples of the CC2, CC3 and TC1 composites lost 34.45%, 20.21% and 57.9% of their mass, respectively. The smallest impact of the aggressive environment on concrete based on sanitary ceramic aggregate could be related to the lowest absorptivity of this aggregate from among ceramic aggregates. Red ceramics, in turn, are characterized by the highest absorptivity, and concrete based on this aggregate is the least resistant to aggressive environments. Both in these and the previous samples, it was noticed that the cause of the destruction, mainly of the cement matrix, was an aggressive environment, although in the case of gravel aggregate, red ceramics and basalt, the aggregate was also deteriorated. The grains made of sanitary ceramics did not deteriorate. Their appearance indicated that the aggregate was a kind of protection (shield) against the chemical deterioration of the matrix due to the aggressive environment. This could also be due to the specific types of bonding between aggregate and cement paste mentioned above, which, apart from adhesion, were defined as mechanical. The destruction of composites, i.e., samples after 240 days of saturation in the solution, is shown in Figure 11.

After 30 days of immersion in the solution, the decrease in the mass of the samples was almost zero. For comparison, in other studies [71], the reduction in weight of recycled concrete (based on concrete waste aggregates) cubes after 30 days of immersion in 3% H_2_SO_4_ was about 5.24%. Gireesh [72], in turn, proved that loss of weight in recycled concrete (based on aluminum dross aggregates) mixes due to acid attack (5% H_2_SO_4_) was in the range of 1.06–2.23%. 

Considering both the first and the second criterion, the research demonstrated the beneficial effect of the presence of ceramic aggregates (in particular derived from sanitary ceramics) on the resistance to aggressive environments. A substitute for traditional aggregate in the form of crushed waste sanitary wares resulted in lower losses of composite mass and higher compressive strength compared to traditional composites. It was therefore concluded that the use of recyclate from the sanitary cullet was the most advantageous for the production of concrete resistant to aggressive environments, and is recommended for the production of sewerage pipes.

There was no relationship between the results of chemical deterioration resistance tests and the results of chemical composition of aggregates tests. Similar chemical composition of recycled aggregates and various images of their destruction showed a greater impact on the examined feature of physical parameters. Features such as glazing of the surface and diversified structure probably had a greater impact on the chemical resistance than the chemical composition of the ceramic aggregates.

## 4. Conclusions

This research presents the possibilities of using three types of ceramics as concrete aggregate. The basic parameters of the aggregates were investigated. The use of recycled aggregates from ceramic construction waste is possible, although the analyses conducted prove that there are many factors which determine the characteristics of the final composite.

A thorough analysis of the obtained results enabled to formulate the following conclusions:

- Recycled ceramic aggregates present relatively lower apparent density and higher absorptivity compared to traditional aggregates (basalt and gravel). Other ceramic aggregates are characterized by higher, but similar, absorptivity to traditional aggregates.
- Red ceramic aggregate is characterized by a greater degree of crushing compared to glazed and sanitary ceramic aggregate, by 205.7% and 439.4%, respectively.
- Depending on the type of recycled aggregate used, the technical properties of the concretes obtained from it are different.
- The consistency of composites with ceramic recycled aggregates was lower than in the case of traditional composites, despite the same cement paste ingredients being used.
- The apparent density of composites with recycled aggregates was lower than in traditional composites; absorptivity of recycled composites was higher than that of traditional composites. All these characteristics result from the examined aggregate features, i.e., low density and high absorptivity.
- The concrete based on sanitary aggregate is characterized by 22% higher flexural strength and 42% higher compressive strength than concrete based on glazed ceramic aggregate and flexural and compressive strength 59% higher than that of concrete based on the red ceramic aggregate.
- The study proved that sanitary ceramic concrete is characterized by greater strength than concrete based on basalt aggregate, by 10.5% and by 5.9% for the compressive and the flexural strength, respectively. The parameters of the concretes made with glazed ceramic can constitute a substitute for gravel concrete. The concretes based on a glazed ceramic aggregate are characterized by similar strength properties to the properties of concrete based on gravel.
- The concrete based on red ceramic aggregate is characterized by the flexural strength lower by 10% and the compressive strength lower by 20% than the strength of the gravel-based concrete.
- The lowest drop in strength after the impact of a chemically aggressive environment was observed in the concrete based on sanitary ceramics aggregate (13.4%). Among the concretes based on traditional aggregates, the lowest compressive strength loss was observed in the basalt concrete (27.8%).
- The smallest weight loss caused by aggressive environment attack was recorded in the concrete based on ceramic sanitary and glazed aggregate (20.2% and 34.5%, respectively, after 120 days of aggressive environment). Among the concretes based on traditional aggregates, the lowest weight loss was observed in the basalt concrete (57.9%).
- The use of sanitary ceramic cullet as aggregate is the most advantageous in the production of the concrete resistant to aggressive environments and is recommended for the production of concrete sewage pipes.

## Figures and Tables

**Table 1:** The chemical composition of the aggregates used in the investigation.

Component	Sand/Gravel	Basalt Grid	Red Ceramics	Glazed Ceramics	Sanitary Ceramics
Unit	[%]				
SiO_2_	16.66	64.84	60.48	54.53	67.63
Al_2_O_3_	7.97	14.22	29.99	34.87	24.05
K_2_O	0.77	2.82	4.83	1.45	3.00
NiO	-	-	2.04	1.58	2.78
Na_2_O	0.94	5.60	1.43	2.36	1.25
Fe_2_O_3_	1.03	-	1.02	1.45	0.55
MgO	40.85	0.55	0.18	1.60	0.36
Mo_2_O_3_	-	-	-	-	0.37
CaO	31.78	11.62	-	-	-
SO_3_	-	0.33	-	-	-

**Table 2:** The results of a study to determine the optimal proportion of ceramic aggregate fraction.

Parameter of Optimization	Unit	Subsequent Tests					
Mass ratio of particle size *A1: **A2	-	1:0	1:0.25	1:0.3	1:0.35	1:0.4	1:0.45
Amount of aggregate taken for testing (A1:A2 by mass)	kg	3:0	3:0.75	3:0.9	3:1.05	3:1.2	3:1.35
Specific density of aggregates mixture	kg/m^3^	2504	2549	2599	2603	2636	2625
Bulk density of aggregates mixture	kg/m^3^	1479	1505.6	1535.1	1537.5	1557	1550.5

*A1—fine ceramic aggregate; **A2—coarse ceramic aggregate.

**Table 3:** A reference recipe for C35/45 concrete.

Component	Unit	Amount
Cement	kg/m^3^	370
CEM I 42.5N—SR 3/NA		
Aggregate:	kg/m^3^	667
Sand-gravel 0/4 mm		
Aggregate:	kg/m^3^	1296
Basalt grit 4/8 mm		
Water	kg/m^3^	139
Admixture ISOFLEX 7130	kg/m^3^	5.6
Silica fume	kg/m^3^	74

**Table 4:** Symbols of the tested concrete recipes.

Symbol of Concrete Recipe	Description
CC1	Ceramic concrete based on red ceramic aggregate
CC2	Ceramic concrete based on glazed ceramic aggregate
CC3	Ceramic concrete based on sanitary ceramic aggregate
TC1	Traditional concrete based on basalt aggregate
TC2	Traditional concrete based on gravel aggregate

**Table 5:** The composition of the solution.

Components	Amount
Tap water	7 dm^3^
Sulfuric acid H_2_SO_4_ (96%)	7 mL
Magnesium sulfate in the form of heptahydrate MgSO_4_·7H_2_O	219.6 g
Ammonium sulfate ${\left({\mathrm{NH}}_{4}\right)}_{2}{\mathrm{SO}}_{4}$	64.2 g

**Table 6:** Ceramic aggregate features compared to other aggregates used in concrete [52]. (*—Data from the authors’ own research).

Type of Aggregate/Feature	Unit	Traditional Aggregate: Sand, Gravel	Basalt Grit	Recycled Ceramic Aggregate		
				Red Ceramics	Glazed Ceramics	Sanitary Ceramics
Specific density	kg/m^3^	2650	2600–3200	1400 *	2200 *	2640 *
Apparent density	kg/m^3^	1800–2000	2500–3100	1000 *	2040 *	2360 *
Compressive strength	MPa	22–45	250–400	5–20	110–360	60–600
Modulus of elasticity	GPa	20–40	56–99	10–30	20–36	40–70
Absorptivity	%	0.6–2.8	0.1–0.4	22.0 *	2.9 *	1.53 *
Degree of crushing	%	8.0 16.0	3.8	48 *	15.7 *	8.9 *

**Table 7:** The consistency of concrete mixes.

Symbols	Average Slump (mm)	Standard Deviation (mm)	Variability Indicator (%)	Consistency Class
CC1	205	2.4	0.86	S4
CC2	200	1.9	0.64	S4
CC3	203	2.2	0.53	S4
TC1	235	1.8	0.91	S5
TC2	230	2.0	0.87	S5

**Table 8:** The apparent density and absorptivity of tested composites

Properties/Symbols	CC1	CC2	CC3	TC1	TC2
Apparent density [kg/m^3^]	2210	22340	22280	23820	25460
Absorptivity [%]	7.01	6.92	6.74	5.58	5.74

**Table 9:** Changes in mass of samples.

Sample Symbol	Percent Increase/Loss of Mass Tested in a Given Time Intervals (%)							
Time Intervals	0 days	14 days	30 days	60 days	90 days	120 days	240 days	360 days
CC1	0	1.44	−0.330	−10.35	−34.20	−78.40	−100	−100
CC2	0	1.33	−0.010	−5.82	−24.90	−34.45	−68.45	−100
CC3	0	1.23	0.0120	−4.30	−21.54	−20.21	−51.60	−100
TC1	0	1.34	0.014	−7.32	−31.44	−57.90	−100	−100
TC2	0	1.14	−0.330	−11.70	−39.21	−70.60	−100	−100
